# Dissipative Particle Dynamics of Nano-Alumina Agglomeration in UV-Curable Inks

**DOI:** 10.3390/polym16182609

**Published:** 2024-09-14

**Authors:** Chunlai Li, Liang Guo, Weihan Zheng

**Affiliations:** 1 Changchun Institute of Optics, Fine Mechanics and Physics, Chinese Academy of Sciences, Changchun 130033, China; 15040873714@163.com (C.L.);; 2 University of Chinese Academy of Sciences, Beijing 100049, China

**Keywords:** dissipative particle dynamics, inkjet printing, particle agglomerate, UV ink

## Abstract

Ultraviolet (UV) ink is a primary type of ink used in additive manufacturing with 3D inkjet printing. However, ink aggregation presents a challenge in nano-inkjet printing, affecting the stability and quality of the printing fluid and potentially leading to the clogging of nanometer-sized nozzles. This paper utilizes a Dissipative Particle Dynamics (DPD) simulation to investigate the aggregation behavior of alumina in a blend of 1,6-Hexanediol diacrylate (HDDA) and Trimethylolpropane triacrylate (TMPTA). By analyzing the effects of solid content, polymer component ratios, and dispersant concentration on alumina aggregation, the optimal ink formulation was identified. Compared to traditional experimental methods, DPD simulations not only reduce experimental costs and time but also reveal particle aggregation mechanisms that are difficult to explore through experimental methods, providing a crucial theoretical basis for optimizing ink formulations. This study demonstrates that alumina ceramic ink achieves optimal performance with a solid content of 20%, an HDDA-to-TMPTA ratio of 4:1, and 9% oleic acid as a dispersant.

## 1. Introduction

**Figure 1:**
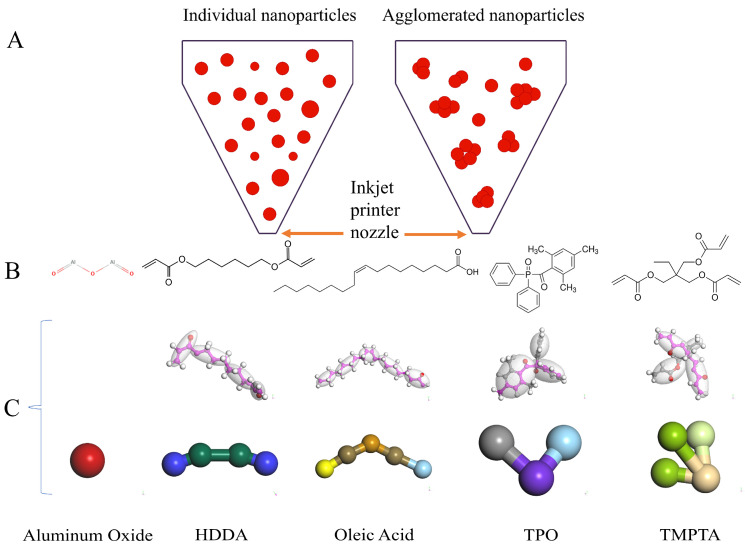
Schematic diagram showing individual and aggregated nanoparticles passing through the nozzle tip (**A**), the chemical structure of each UV ink component (**B**), and the coarse-grained structural model (**C**).

With the rapid advancement of 3D inkjet printing technology, the issue of particle agglomeration in alumina ceramic inks, which can lead to nozzle clogging, has become particularly prominent [1]. At the nanoscale, nanoparticles tend to aggregate, flocculate, and settle, forming strong flocculated states. Research indicates that for additive manufacturing at the nanoscale, the particle size should be approximately 1/100 of the nozzle diameter to prevent clogging and printing defects [2]. As shown in Figure 1A, agglomeration within the printing material can lead to nozzle blockage. Experimental studies of nanoparticle ink agglomeration require substantial resources, including costly equipment and time-consuming sample preparation and measurement processes.

In 1992, Hoogerbrugge and Koelman [3] introduced a mesoscopic simulation method known as Dissipative Particle Dynamics (DPD). To overcome the limitations of traditional experimental methods, this study aims to use DPD simulations to explore factors influencing the aggregation of alumina ceramic ink particles, with a particular focus on the roles of monomers and additives. Compared to molecular dynamics simulations, DPD employs a coarse-grained model and softer inter-particle interaction potentials, while preserving momentum-conserving thermal baths. Additionally, DPD can simulate larger systems over shorter timescales, reducing computational costs and time. As a result, it has become a valuable tool for studying mesoscopic assembly and phase behavior in polymer systems.

Compared to experimental research, DPD simulations, conducted on computers, save considerable experimental costs and time. By adjusting model parameters and initial conditions, DPD simulations can generate large datasets in less time, enabling in-depth analysis changes and trends in aggregation behavior. Additionally, DPD simulations allow for the precise control and adjustment of model parameters, providing detailed information on the internal structure, particle interactions, and dynamics of aggregates. This enables a comprehensive understanding of particle positions, velocities, and energy states, revealing the mechanisms of aggregate formation and evolution, which can be challenging to explore experimentally due to technical or cost limitations. DPD simulations enable researchers to identify and analyze potential aggregation mechanisms in nanoparticle inks, such as inter-particle interactions and the roles of dispersants. This detailed mechanistic study can aid in optimizing ink formulations and processes, thus improving printing quality and performance.

DPD simulations are based on integrating Newton’s equations of motion, utilizing a series of beads to represent clusters of atoms or molecules, thereby simulating mesoscopic fluid dynamics systems, which are especially suited for colloidal suspensions [4,5,6,7,8]. DPD simulations have been successfully applied to various complex systems, including polymers, colloids, phospholipids, liquids, and crystalline proteins, producing significant results [9,10,11,12,13]. For instance, Qinghua Wei et al. [14] utilized DPD simulations to investigate the agglomeration behavior of nano-silica in PVA/PAM blend hydrogels, examining factors like nano-silica content, monomer composition ratios, temperature, and shear rate. In contrast, Suphanat Aphinyan [15,16] employed DPD simulations to model UV inks, exploring the impact of different monomer types and their proportions on agglomeration morphology and the effectiveness of surfactants in controlling nanodrop formation, using Multibody Dissipative Particle Dynamics (MDPD) to predict nanodrop formation in nanoscale nozzles [17].

However, in alumina ceramic inks, the agglomeration of alumina particles primarily causes nozzle blockage. Therefore, this study focuses on the effects of monomers and other additives affecting alumina agglomeration. The alumina ceramic ink primarily consists of alumina, monomers (1,6-Hexanediol diacrylate (HDDA) and Trimethylolpropane triacrylate (TMPTA)), a photoinitiator (Diphenyl (2,4,6-trimethylbenzoyl) phosphine oxide (TPO)), and a dispersant (Oleic acid), as shown in Figure 1B. HDDA and TMPTA are monomers with multiple acrylate groups that can participate in free radical polymerization. These acrylate groups provide numerous double bonds that can cross-link with other monomers. The surface of alumina may contain hydroxyl groups (-OH), which can undergo esterification reactions with the acrylate groups. During polymerization, the multiple acrylate groups of HDDA and TMPTA can cross-link on the alumina surface, creating a network structure. In the coarse graining process, we generate various beads from the atomic model, with beads of different colors representing different block copolymers or homopolymers in the statistical chain segments as shown in Figure 1C. Using DPD simulation methods, the mesoscopic morphology and rheological properties of alumina particles in a blend suspension with HDDA and TMPTA as the primary polymers were revealed. The focus was on optimizing the particle aggregation conditions of alumina ceramic inks to determine the optimal ink formulation.

## 2. Materials and Methods

### 2.1. Dissipative Particle Dynamics Method

In the DPD method, interactions between beads occur only within a cut-off radius $r_{c}$, which defines the system’s length scale. When the distance between two beads exceeds 1 [18,19], their interaction force is zero. This approach reduces computational time and significantly improves efficiency. The motion of each bead in DPD is governed by Newton’s equations of motion as follows:

(1) $$\underset{j\neq i}{\sum F_{\mathrm{ij}}}=\frac{d_{P_{i}}}{d_{t}}$$

(2) $$v_{i}=\frac{{\mathrm{dr}}_{i}}{\mathrm{dt}}$$

Here, $F_{\mathrm{ij}}^{C}$ represents the total force exerted on bead i by bead j, while $p_{i}$, $v_{i}$, and $r_{i}$ denote the momentum, velocity, and position vector of bead i, respectively. Developed by Espanol and Warren [20] in 1995, the total force in DPD, as expressed in Equation (1), is the sum of three types of forces acting on each bead:

(3) $$F_{\mathrm{ij}}=F_{\mathrm{ij}}^{C}+F_{\mathrm{ij}}^{D}+F_{\mathrm{ij}}^{R}$$

The conservative force $F_{\mathrm{ij}}^{C}$ represents the repulsive force between beads, the dissipative force $F_{\mathrm{ij}}^{D}$ accounts for the viscous force that resists relative motion and reduces the velocity difference between beads, and the random force $F_{\mathrm{ij}}^{R}$ denotes the stochastic fluctuation force on the beads. These three forces are typically expressed as follows:

(4) $$F_{\mathrm{ij}}^{C}=\omega^{c}\left(r_{\mathrm{ij}}\right)e_{\mathrm{ij}}$$

(5) $$F_{\mathrm{ij}}^{D}=-\gamma \omega^{D}\left(r_{\mathrm{ij}}\right)\left[\upsilon_{\mathrm{ij}}\cdot e_{\mathrm{ij}}\right]e_{\mathrm{ij}}$$

(6) $$F_{\mathrm{ij}}^{R}=\sigma \omega^{R}\left(r_{\mathrm{ij}}\right)\theta_{\mathrm{ij}}e_{\mathrm{ij}}$$

Here, $r_{\mathrm{ij}}$ represents the linear distance between beads i and j, and $e_{\mathrm{ij}}$ indicates the relative direction between the two beads. In Equation (5), γ denotes the dissipation coefficient, with the negative sign indicating that the force is directed opposite to the relative motion of the beads, while $\vec{\upsilon_{\mathrm{ij}}}=\vec{\upsilon_{i}}-\vec{\upsilon_{j}}$ represents the relative velocity between the beads. In Equation (6), σ is the random force coefficient, and $\sigma^{2}=\frac{2\gamma \kappa_{B}T}{m}$ relates σ and γ, where $K_{B}$ is the Boltzmann constant and T is the system temperature. This relationship couples the dissipative and random forces. $\theta_{\mathrm{ij}}$ is a random variable representing random disturbances between the beads, and it satisfies Equation (7). $\omega^{C}$, $\omega^{D}$, and $\omega^{R}$ denote the conservative, dissipative, and random force weight functions, respectively.

(7) $$\begin{array}{c} \left\langle \theta_{\mathrm{ij}}\left(t\right)\right\rangle =0\\ \left\langle \theta_{\mathrm{ij}}(t)\theta_{\mathrm{kl}}(t^{\prime })\rangle =(\delta_{\mathrm{ik}}\delta_{\mathrm{jl}}+\delta_{\mathrm{il}}\delta_{\mathrm{jk}})\delta (t-t^{\prime }\right) \end{array}$$

(8) $$\omega^{C}\left(r_{\mathrm{ij}}\right)=\{\begin{array}{c} a_{\mathrm{ij}}\left(1-\frac{r_{\mathrm{ij}}}{r_{c}}\right),\;\;\;\;\;\;r_{\mathrm{ij}}<r_{c}\\ 0\;\;\;\;\;\;\;\;\;\;\;,\;\;\;\;\;\;r_{\mathrm{ij}}\geq r_{c} \end{array}$$

(9) $$\omega^{D}\left(r_{\mathrm{ij}}\right)={\left[\omega^{R}\left(r_{\mathrm{ij}}\right)\right]}^{2}=\{\begin{array}{c} {\left(1-r_{\mathrm{ij}}/r_{c}\right)}^{2}\;\;,\;\;\;\;\;r_{\mathrm{ij}}<r_{c}\\ 0\;\;\;\;\;\;\;\;\;\;\;\;\;\;,\;\;\;\;\;r_{\mathrm{ij}}\geq r_{c} \end{array}$$

Here, $a_{\mathrm{ij}}$ represents the repulsion parameter between beads i and j, which can be calculated using the Flory–Huggins $\left(\chi_{\mathrm{ij}}\right)$ parameter. The Flory–Huggins $\left(\chi_{\mathrm{ij}}\right)$ parameter itself can be defined through the solubility parameter δ and the molar volume $V_{\mathrm{ref}}$ [21]. In this study, the solubility parameter δ and molar volume $V_{\mathrm{ref}}$ of the beads were determined through calculations, with specific values provided in Table 1. The calculated results for $\chi_{\mathrm{ij}}$ and $a_{\mathrm{ij}}$ are listed in Table 2 and Table 3, respectively, according to Equations (11) and (10).

(10) $$a_{\mathrm{ij}}=a_{\mathrm{ii}}+3.27\chi_{\mathrm{ij}}$$

(11) $$\chi_{\mathrm{ij}}={\left(\delta_{i}-\delta_{j}\right)}^{2}V_{\mathrm{ref}}/K_{B}T$$

In the case of interactions among identical DPD beads:

(12) $$a_{\mathrm{ii}}=75K_{B}T/\rho$$

Here, ρ represents the bead density. When P is 3 and the system energy $K_{B}T$ decreases to 1, the value of $a_{\mathrm{ii}}$ is 25.

### 2.2. DPD Model Parameters

**Figure 2:**
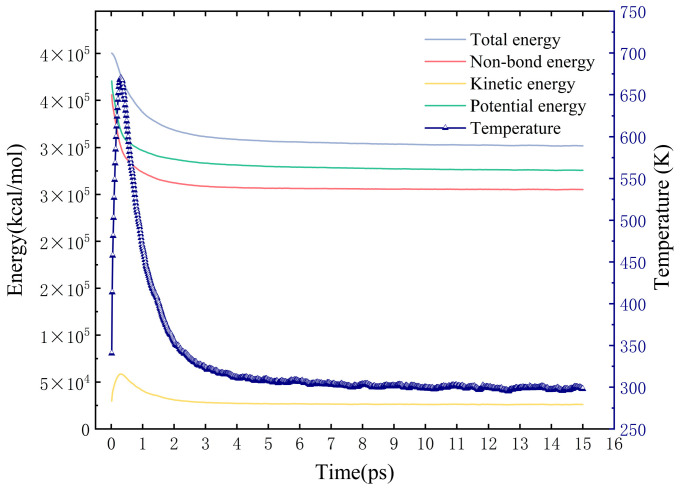
The curves of energy and temperature (represented in navy blue) show the transition from the initial state to the equilibrium state over 15 ps in the DPD simulation.

The simulation utilized a cubic cell with dimensions of 100 × 100 × 100 Å^3^ and employed periodic boundary conditions. The simulation comprised approximately 27,000 DPD beads, with additional key parameters detailed in Table 4. After 30,000 simulation steps, the total simulation time amounted to 15 ps. To ascertain if the system had achieved equilibrium, we monitored its energy and temperature over time. As depicted in Figure 2, which illustrates a representative example from all simulations, the system had indeed reached equilibrium, with the temperature stabilizing around 298 K.

## 3. Results and Discussions

### 3.1. Influence of Monomer Proportion

**Figure 3:**
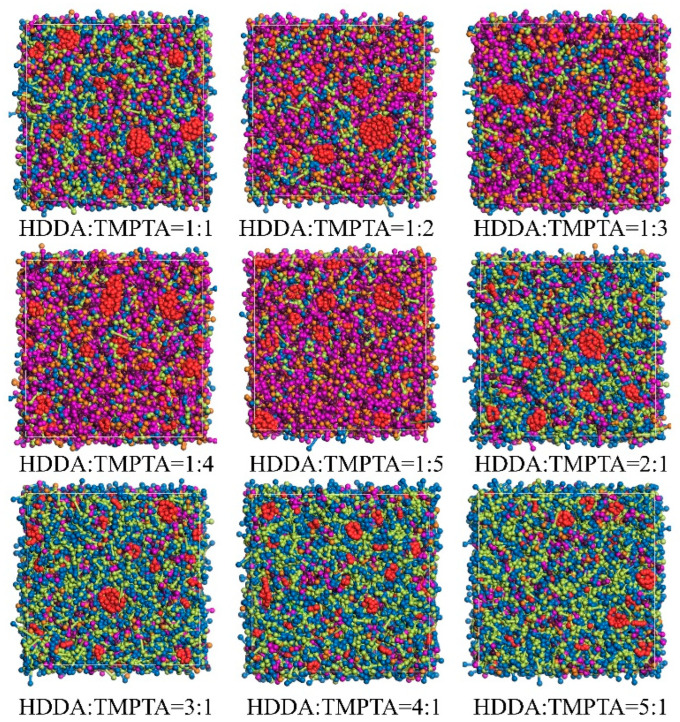
Aggregation of alumina (red) at varying monomer proportions.

Monomers, often referred to as functional monomers, are organic small molecules containing polymerizable functional groups that play a crucial role in light-cured alumina ceramic inks. Currently, the most widely used monomer in alumina ceramic slurry formulations is HDDA, which is typically used in conjunction with TMPTA. Monomers not only adjust the viscosity of the slurry system but also participate in the light-curing process, influencing the various properties of the light-cured product. Therefore, selecting the optimal monomer ratio is crucial in designing light-curing slurry formulations. To investigate how monomer component ratios affect the agglomeration behavior of nano-alumina, a simulation system was employed with only monomers and alumina added, and nine comparative simulations were conducted. In these simulations, the number of alumina beads was kept constant at 20, while the monomer ratios (HDDA:TMPTA) were varied as 5:1, 4:1, 3:1, 2:1, 1:1, 1:2, 1:3, 1:4, and 1:5. When equilibrium was reached, different HDDA-to-TMPTA ratios resulted in varying agglomeration morphologies, as shown in Figure 3.

Figure 3 demonstrates that the agglomeration morphology of alumina is affected by the concentration ratio of HDDA to TMPTA. The added monomers, and their respective polymers, aggregate, resulting in varying degrees of alumina agglomeration. This is because monomers share similar electrostatic properties, while the alumina ceramic powder surfaces, which are hydrophilic due to hydroxyl groups, tend to clump together, hindering even dispersion in the suspension. An inappropriate monomer ratio can lead to excessive aggregation or dispersion of the monomers, thus affecting the morphology and size of alumina agglomerates. Visual inspection of the simulation results reveals that at an HDDA:TMPTA ratio of 4:1, the alumina agglomerates exhibit the smallest size and the most uniform distribution, which is consistent with Zhangwei Chen’s findings [22].

### 3.2. The Proportion of Dispersant Affects the Amount of Dispersant Added

**Figure 4:**
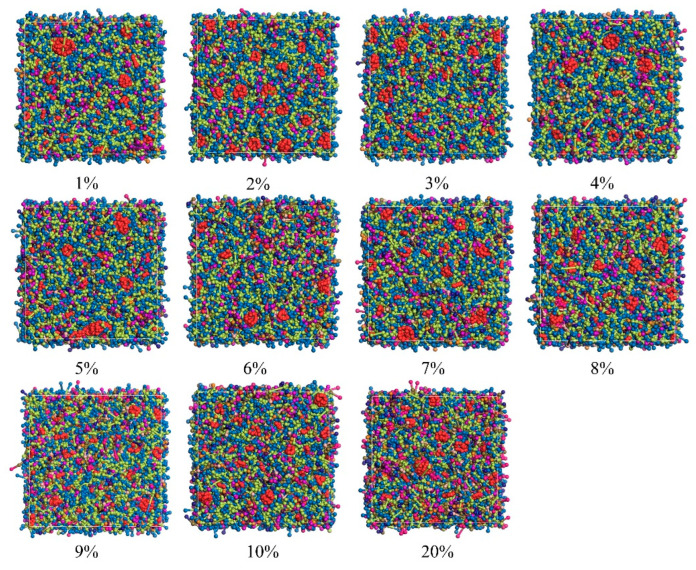
Aggregation of alumina (red) at different dispersant ratios. (1%, 2%, 3%, 4%, 5%, 6%, 7%, 8%, 9%, 10%).

The surface of alumina particles is covered with numerous hydroxyl groups, which impart a charge to the particles and causes them to interact through van der Waals forces, resulting in electrostatic attraction. This attraction leads to significant flocculation of the powder particles, increasing the slurry’s viscosity at high solid content and causing uneven dispersion of the powder. Consequently, large agglomerates form, blocking the inkjet printer nozzles and failing to meet the requirements for photopolymer printing. To address this issue, dispersants were introduced into the alumina ceramic ink as additives in alumina ceramic ink. Zhu, Wusheng et al. [23] used molecular dynamics simulations to study the interactions between surfactants and polymers for the prevention and stabilization of small crystal agglomerates. Laser diffraction experiments combined with simulations revealed that surfactants play a crucial role in controlling the crystal size in suspensions, keeping it well below 4.7 μm. Dispersants utilize steric stabilization mechanisms, where molecules adsorb onto the powder surface and generate repulsive forces when particles overlap in space, preventing the agglomeration of alumina powder particles. To investigate the effect of oleic acid dispersants on alumina ceramic ink, we designed 11 models of alumina ceramic ink with varying dispersant ratios: 1%, 2%, 3%, 4%, 5%, 6%, 7%, 8%, 9%, 10%, and 20%, while maintaining the polymer ratio constant at HDDA: TMPTA = 4:1. Figure 4 displays some of the simulation results.

**Figure 5:**
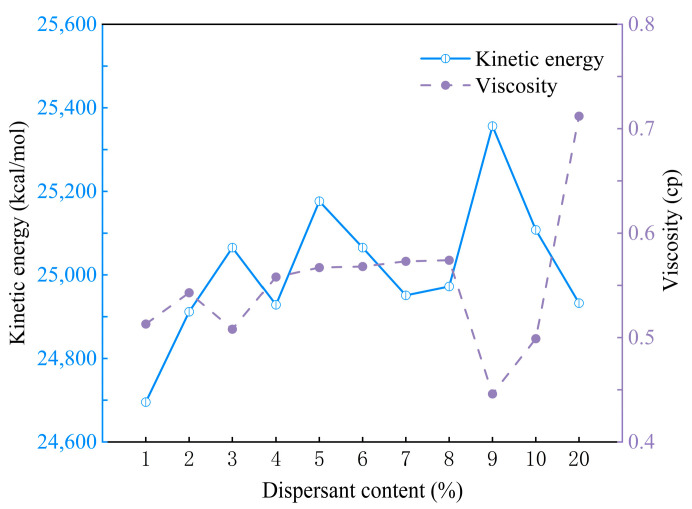
Kinetic energy and viscosity of alumina ceramic inks with different dispersant ratios.

According to the research findings, the amount of dispersant significantly impacts the viscosity and agglomeration behavior of the ink system. Specifically, there is an optimal amount of dispersant, corresponding to the point of minimum viscosity of the slurry [24]. When the dispersant amount is insufficient, it fails to adequately cover and encapsulate the surface of the alumina particles, leading to inadequate reduction in inter-particle attraction and resulting in agglomeration and flocculation. Conversely, when the dispersant amount exceeds the maximum adsorption capacity, excessive dispersant increases the slurry’s viscosity. Simulation results show that the viscosity of the ink system initially decreases and then increases with increasing dispersant concentration, as observed in Figure 5 (purple curve). Specifically, at a shear rate of 0.1 1/PS, the ink system with 9% dispersant exhibits the lowest viscosity. Combining this with the dispersion of alumina particles shown in Figure 4, it indicates that an appropriate amount of dispersant effectively suppresses agglomeration and enhances the flow properties of the ink. Moreover, in addition to viscosity and agglomeration morphology, kinetic energy is also a good indicator for assessing agglomeration probability. High kinetic energy is typically associated with reduced agglomeration [25]. According to the results presented in Figure 5 (navy blue curve), the ink system with 9% dispersant has the highest kinetic energy (25,356.335 kcal/mol), indicating the lowest agglomeration probability.

### 3.3. Influence of Solid Phase Content 

**Figure 6:**
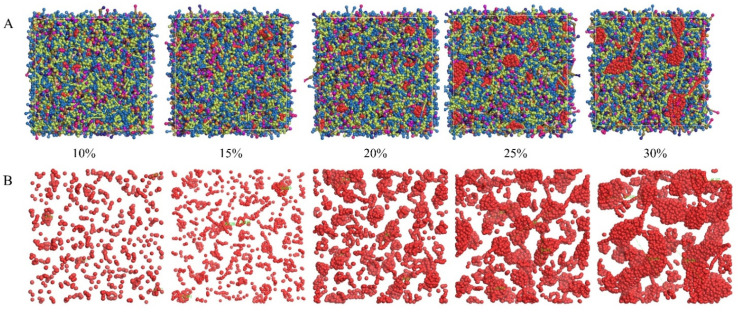
DPD simulation results of different solid phase contents (left to right: 10%, 15%, 20%, 25%, and 30% of the mesoscopic morphology; (**A**) represents all beads, where red represents alumina beads, and (**B**) represents only alumina beads).

To prepare high-performance alumina ceramic inks, the ink must have a high solid content, good stability, and excellent rheological properties to ensure that the resulting ceramic achieves complex shapes, low sintering shrinkage, uniform volume density, and stable performance. Specific requirements for solid content are influenced by factors such as ink formulation, printing medium, and equipment. Typically, a low solid content can result in incomplete curing of the ink on the printing medium, affecting the image quality and durability of the finished product. Conversely, a high solid content can lead to issues such as particle agglomeration, excessively high viscosity, and nozzle clogging. To investigate the impact of solid content on ink agglomeration, we established DPD models of alumina ceramic inks with varying alumina contents (10%, 15%, 20%, 25%, and 30%) and analyzed simulation results. The relevant simulation results are shown in Figure 6.

**Figure 7:**
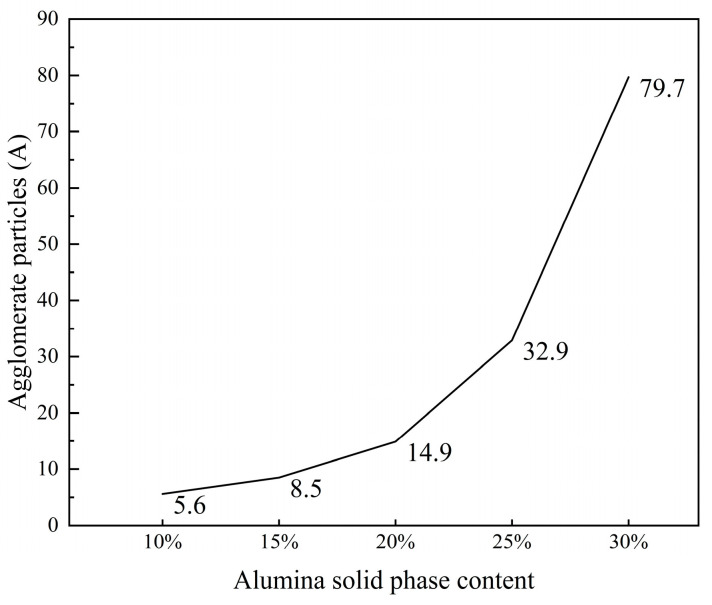
Relation between solid phase content and agglomeration degree of alumina particles.

An increase in solid content significantly affects the dynamic mechanical properties of the ink system. Under high solid content conditions, the ink system tends to form larger aggregates due to aggregation phase transition. As the solid content increases, the concentration of alumina particles in the ink rises, enhancing intermolecular interactions and reducing the free movement space between molecules. This results in the denser cross-linking of polymer molecules, forming a more stable polymer network structure that facilitates clustering, as shown in Figure 6. However, inks with lower solid content exhibit reduced stability. Although printing is feasible, it becomes difficult for the printer to accurately control the droplet position, affecting subsequent processes and the quality of inkjet-printed products [26]. Additionally, we measured the average particle size of the four largest alumina aggregates at various solid contents, as shown in Figure 7. The results indicate a positive correlation between the solid content of inkjet ceramic inks and agglomeration degree. When the solid content exceeds 20%, the increase in the particle size of alumina aggregates becomes more significant, reducing the value of further research.

Therefore, to ensure that alumina ceramic ink maintains high solid content, good stability, and excellent rheological properties—thereby ensuring that the formed ceramics have complex shapes, low sintering shrinkage, uniform volume density, and stable performance—while avoiding nozzle clogging due to alumina agglomeration at high solid contents, it is reasonable to choose a solid content of 20% for subsequent studies. This solid content balances ink flowability and ejection performance while effectively controlling agglomeration degree, ensuring the stability and quality of the printing process.

## 4. Conclusions

This paper establishes a DPD model for UV-curable alumina ceramic inks employed in inkjet printing to investigate the interaction mechanisms of different additives in UV-curable alumina ceramic inks. It reveals alterations in the mesoscopic morphology of alumina particles under various formulations. This study aims to optimize the particle aggregation conditions of alumina ceramic ink and systematically examines key factors affecting alumina aggregation morphology by controlling a single variable. Numerical simulations show that an ink with 20% alumina, 9% dispersant, and an HDDA:TMPTA ratio of 4:1 exhibits the best performance in terms of uniformity, aggregate size, and dispersibility. Notably, oleic acid is crucial for in reducing aggregation, controlling the morphology of the monomer blend, and enhancing structural uniformity. In conclusion, the dispersibility of alumina in ceramic ink is a key factor in preventing nanometer nozzle clogging and ensuring high-quality printing results.

## Figures and Tables

**Table 1:** Solubility parameters δ and molar volume $V_{\mathrm{ref}}$ of DPD beads.

	$\delta \left({MPa}^{1/2}\right)$	$V_{\mathrm{ref}}\left({cm}^{3}/mol\right)$
TPO-C_6_H_5_	20.87	385.08
TPO-C_10_H_11_O	19.71	779.44
TPO-C_6_H_5_OP	22.26	1175.16
HDDA-C_3_H_3_O	20.39	779.44
HDDA-C_3_H_6_O	17.98	218.09
AL_2_O_3_	141.19	42.98
TMPTA-C_5_H_5_O_2_	18.90	402.08
TMPTA-C_2_H_5_	18.66	161.99
TMPTA-C_4_H_5_O_2_	19.42	377.72
Oleic acid-C_3_H_7_	17.98	258.79
Oleic acid-C_4_H_8_	18.52	326.17
Oleic acid-C_4_H_6_	20.44	269.53
Oleic acid-C_3_H_5_O_2_	26.86	294.87

**Table 2:** Flory–Huggins $\left(\chi_{\mathrm{ij}}\right)$ parameters of DPD beads.

	TPO-C_6_H_5_	TPO-C_10_H_11_O	TPO-C_6_H_5_OP	HDDA-C_3_H_3_O	HDDA-C_3_H_6_O	AL_2_O_3_	TMPTA-C_5_H_5_O_2_	TMPTA-C_2_H_5_	TMPTA-C_4_H_5_O_2_	Oleic Acid-C_3_H_7_	Oleic Acid-C_4_H_8_	Oleic acid-C_4_H_6_	Oleic Acid-C_3_H_5_O_2_
TPO-C_6_H_5_	0.00												
TPO-C_10_H_11_O	0.34	0.00											
TPO-C_6_H_5_OP	0.67	2.79	0.00										
HDDA-C_3_H_3_O	0.06	0.16	1.50	0.00									
HDDA-C_3_H_6_O	1.10	0.66	5.61	0.56	0.00								
AL_2_O_3_	832.19	2672.08	3793.55	276.19	872.53	0.00							
TMPTA-C_5_H_5_O_2_	0.67	0.17	3.91	0.39	0.12	1465.35	0.00						
TMPTA-C_2_H_5_	0.59	0.23	3.82	0.21	0.04	677.56	0.01	0.00					
TMPTA-C_4_H_5_O_2_	0.35	0.02	2.76	0.16	0.27	1373.56	0.05	0.07	0.00				
Oleic acid-C_3_H_7_	1.16	0.68	5.77	0.66	0.00	1008.56	0.12	0.04	0.29	0.00			
Oleic acid-C_4_H_8_	0.86	0.35	4.63	0.50	0.03	1223.08	0.02	0.00	0.12	0.04	0.00		
Oleic acid-C_4_H_6_	0.03	0.12	1.05	0.00	0.65	1003.20	0.35	0.30	0.15	0.70	0.49	0.00	
Oleic acid-C_3_H_5_O_2_	5.38	12.10	6.86	5.44	8.90	972.25	9.72	6.77	8.21	9.61	9.52	5.12	0.00

**Table 3:** Repulsive force parameters of DPD beads $a_{\mathrm{ij}}$.

	TPO-C_6_H_5_	TPO-C_10_H_11_O	TPO-C_6_H_5_OP	HDDA-C_3_H_3_O	HDDA-C_3_H_6_O	AL_2_O_3_	TMPTA-C_5_H_5_O_2_	TMPTA-C_2_H_5_	TMPTA-C_4_H_5_O_2_	Oleic Acid-C_3_H_7_	Oleic Acid-C_4_H_8_	Oleic Acid-C_4_H_6_	Oleic Acid-C_3_H_5_O_2_
TPO-C_6_H_5_	25.00												
TPO-C_10_H_11_O	26.12	25.00											
TPO-C_6_H_5_OP	27.18	34.13	25.00										
HDDA-C_3_H_3_O	25.19	25.52	29.91	25.00									
HDDA-C_3_H_6_O	28.61	27.15	43.34	26.82	25.00								
AL_2_O_3_	2746.26	8762.70	12429.91	928.15	2878.18	25.00							
TMPTA-C_5_H_5_O_2_	27.18	25.56	37.79	26.28	25.38	4816.70	25.00						
TMPTA-C_2_H_5_	26.92	25.75	37.49	25.70	25.12	2240.63	25.02	25.00					
TMPTA-C_4_H_5_O_2_	26.15	25.07	34.04	25.52	25.88	4516.53	25.15	25.22	25.00				
Oleic acid-C_3_H_7_	28.81	27.23	43.87	27.16	25.00	3322.99	25.40	25.14	25.94	25.00			
Oleic acid-C_4_H_8_	27.82	26.13	40.13	26.65	25.11	4024.46	25.08	25.01	25.41	25.12	25.00		
Oleic acid-C_4_H_6_	25.08	25.40	28.44	25.00	27.12	3305.46	26.14	25.99	25.49	27.30	26.59	25.00	
Oleic acid-C_3_H_5_O_2_	42.61	64.55	47.43	42.78	54.12	3204.27	56.79	47.15	51.85	56.43	56.13	41.76	25.00

**Table 4:** Key parameters of DPD simulation.

Physical Quantities	Symbols	Value (DPD Unit)
Cutoff radius	$r_{c}$	1.0
Time step	Δt	0.05
Random force coefficient	σ	6
Dissipative force coefficient	γ	18
Particle mass	M	1
Spring coefficient		4.0
System energy	$\kappa_{B}T$	1.0

## Data Availability

The original contributions presented in the study are included in the article. Further inquiries can be directed to the corresponding author/s.
